# Design, Synthesis and Biological Evaluation of Tasiamide Analogues as Tumor Inhibitors

**DOI:** 10.3390/md12042308

**Published:** 2014-04-22

**Authors:** Wei Zhang, Tiantian Sun, Zhenhua Ma, Yingxia Li

**Affiliations:** Department of Medicinal Chemistry, School of Pharmacy, Fudan University, 826 Zhangheng Road, Shanghai 201203, China; E-Mails: zhangw416@fudan.edu.cn (W.Z.); tiantiansunchina@gmail.com (T.S.); zhenhua_ma2@163.com (Z.M.)

**Keywords:** tasiamide, analogues, synthesis, cytotoxicity, marine peptide

## Abstract

Eighteen analogues of the marine cytotoxic linear peptide tasiamide were designed, synthesized and screened for their inhibitory activities against the growth of human nasopharyngeal carcinoma (KB) and human non-small cell lung tumor (A549) cell lines. The results indicated that minor modifications of the *C*-terminuswith aromatic groups were tolerated, with the IC_50_ values between 1.29 and 12.88 μM against these two cancer cell lines. Truncation, minor modifications at the *N*-terminus or elimination of the *N*-methyl groups in *N*-Me-d-Gln and/or *N*-Me-d-Phe residues resulted in inactive analogues.

## 1. Introduction

**Figure 1:**
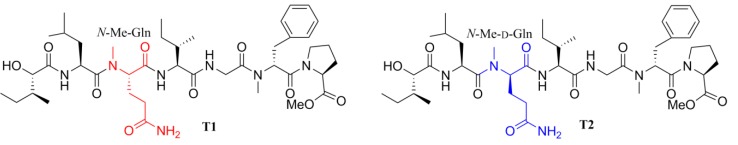
Previously assumed (**T1**) and revised (**T2**) structures of tasiamide.

Marine cyanobacteria are a well-known prolific source of novel bioactive compounds [1,2,3], most of which are linear or cyclic (depsi)peptides with distinctive structures. Tasiamide (Figure 1) is a linear peptide isolated from the marine cyanobacterium *Symploca* sp. in 2002, which showed moderate cytotoxicity against human nasopharyngeal carcinoma (KB) and human colon carcinoma (LoVo) cell lines with IC_50_ values of 0.48 and 3.47 μg/mL, respectively [4]. The first total synthesis of tasiamide was reported by our group [5] in 2008. The result indicated that the previously assumed *N*-Me-l-glutamine residue should be *N*-Me-d-glutamine by comparing the physical data of the synthetic and natural products (^1^H-NMR, ^13^C-NMR and the optical rotation). As a part of our ongoing efforts to find bioactive marine peptides [6,7,8], herein we report the design, synthesis and biological evaluation of some analogues of this natural product.

## 2. Results and Discussion

### 2.1. Chemistry

**Figure 2:**
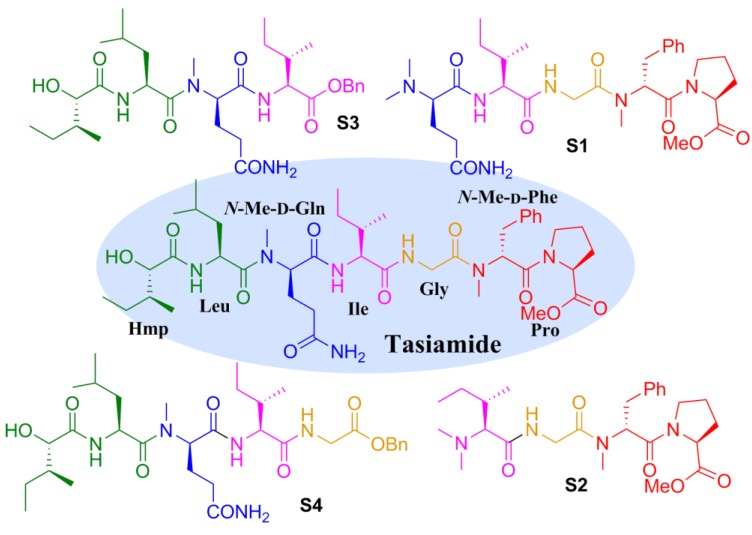
Structures of the designed truncated analogues of tasiamide.

Tasiamide is an acyclic peptide composed of six amino acid residues and an α-hydroxy acid (2-hydroxy-3-methylpentanoic acid (Hmp)). In order to determine the minimal active fragment of this natural product, we decided to design and prepare some truncated analogues (Figure 2). Truncating the Hmp-Leu residues or the Hmp-Leu-*N*-Me-d-Gln residues from the *N*-terminus of tasiamide gave analogues **S1** and **S2**, respectively. Truncating the *N*-Me-d-Phe-Pro residues or Gly-*N*-Me-d-Phe-Pro residues from the *C*-terminus resulted in analogues **S3** and **S4**, respectively.

**Scheme 1:**
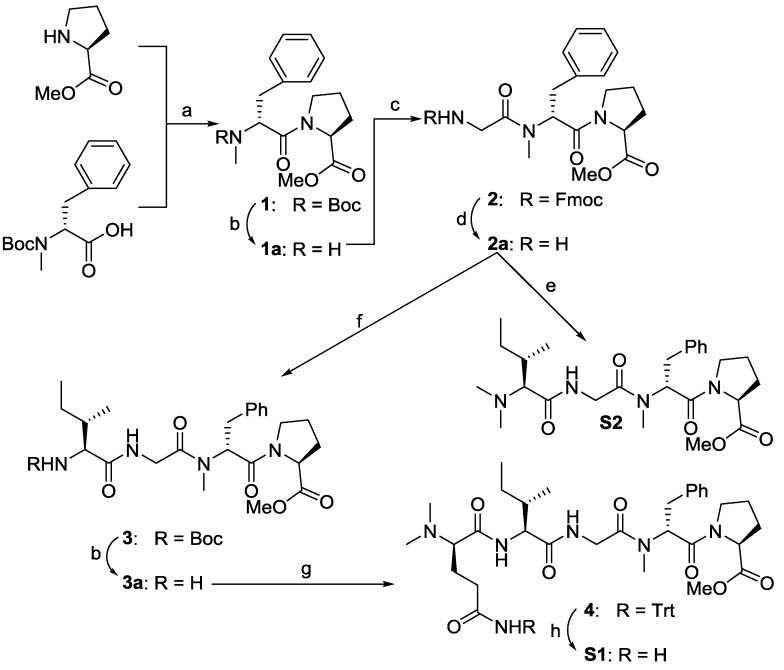
Reagents and conditions: (**a**) 1-ethyl-3-(3-dimethylaminopropyl)-carbodiimide hydrochloride/1-hydroxy-7-azabenzotriazole (EDC/HOAt), NaHCO_3_, dichloromethane (DCM), 12 h, 97%; (**b**) 4 mol/L HCl/EtOAc; (**c**) Fmoc-Gly-OH, EDC/HOAt, NaHCO_3_, 12 h, 77%; (**d**) diethylamine (DEA), CH_3_CN; (**e**) *N*,*N*-Me_2_-Ile-OH, EDC/HOAt, diisopropylethylamine (DIEA), DCM, 24%; (**f**) Boc-Ile-OH, EDC/HOAt, NaHCO_3_, 73%; (**g**) *N*,*N*-Me_2_-d-Gln(Trt)-OH, EDC/HOAt, 95%; (**h**) trifluoroacetic acid (TFA), DCM, 97%.

The preparation of these four truncated analogues are shown in Scheme 1. Dipeptide **1** was prepared by coupling commercially available reagents H-Pro-OMe and Boc-*N*-Me-d-Phe-OH using 1-ethyl-3-(3-dimethylaminopropyl)-carbodiimide hydrochloride/1-hydroxy-7-azabenzotriazole (EDC/HOAt) in dichloromethane (DCM). Removal of the Boc protecting group yielded **1a**, which was used directly in the next cycle of coupling, giving the corresponding tripeptide **2** in good yield. Next, the *N*-Fmoc of **2** was removed under a mild condition by diethylamine (DEA), which was coupled with *N*,*N*-Me_2_-Ile-OH, leading to the desired analogue **S2**. On the other hand, the free amine **2a** was coupled with Boc-Ile-OH, giving tetrapeptide **3** in a 73% yield. After revealing the amino group of compound **3**, *N*,*N*-Me_2_-d-Gln(Trt)-OH was coupled with **3a** to give the fully protected pentapeptide **4** in a yield of 95%. Removal of the Trt group under an acidic condition gave the desired truncated analogue **S1** in a 97% yield.

**Scheme 2:**
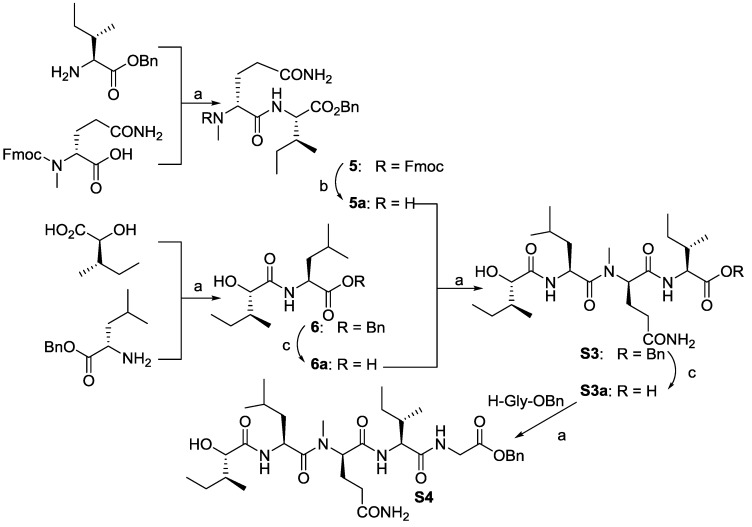
Reagents and conditions: (**a**) EDC/HOAt, *N*-methylmorpholine (NMM), DCM, 12h, 92% for **5**, 91% for **6**, 85% for **S3**, 60% for **S4**; (**b**) DEA/CH_3_CN; (**c**) Pd-C, H_2_, EtOAc.

As shown in Scheme 2, dipeptides **5** and **6** were conveniently synthesized from commercially available amino acids. Deprotections of **5** with DEA and **6** through Pd-mediated hydrogenation liberated the amine and carboxylic acid, respectively, and was subsequently coupled to afford **S3**. Repeating similar procedures, compound **S3a** with carboxylic acid was coupled with H-Gly-OBn to yield target **S4**.

**Figure 3:**
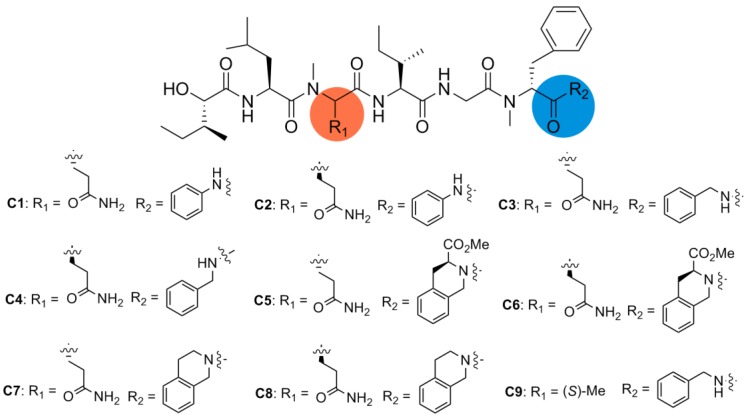
Structures of analogues **C1**–**C9**.

**Scheme 3:**
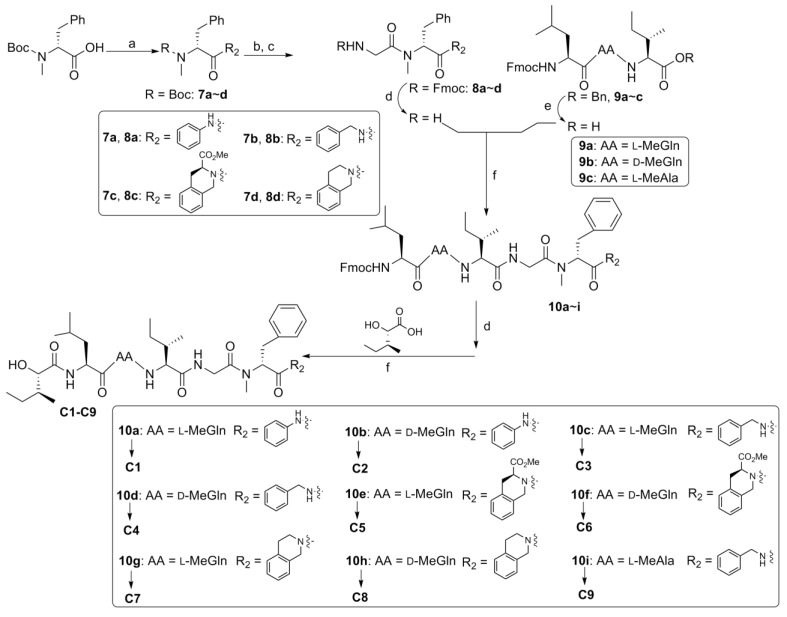
Reagents and conditions: (**a**) R_2_H, EDC/HOAt, NaHCO_3_, 57%~95%; (**b**) HCl/EtOAc; (**c**) Fmoc-Gly-OH, EDC/HOAt, NaHCO_3_, 52%~99%; (**d**) DEA/CH_3_CN; (**e)** H_2_, Pd-C, EtOAc; (**f**) EDC/HOAt. AA = amino acid residue.

In order to determine the effect of *C*-terminus modifications, several full-length analogues were designed, with Pro residue being replaced by an amino group having different aromatic groups (**C1**–**C9**, Figure 3). Starting from Boc-*N*-Me-d-Phe-OH, the desired compounds were obtained by following the standard solution phase peptide synthesis procedure (Scheme 3).

**Scheme 4:**
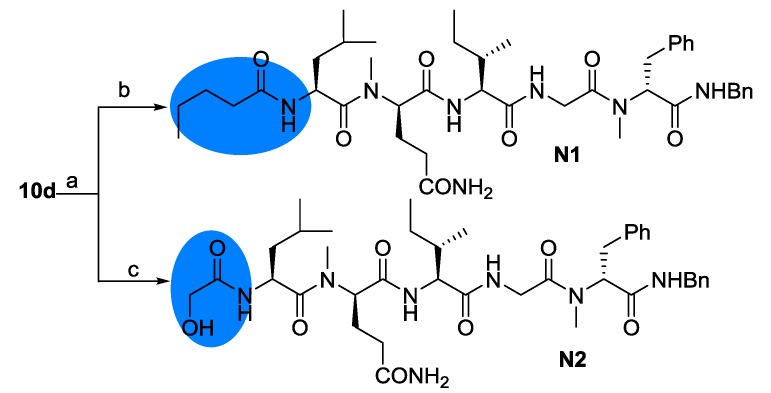
Synthesis of analogues with modification on the *N*-terminus. Reagents and conditions: (**a**) DEA/CH_3_CN; (**b**) EDC/HOAt, pentanoic acid, 17%; (**c**) EDC/HOAt, hydroxyacetic acid, 26%.

In order to investigate the role of the Hmp residue on the *N*-terminus, two analogues were designed. The α-hydroxyl or *sec*-butyl group was truncated to afford **N1** or **N2**, respectively (Scheme 4). With the pentapeptide fragment **10d** in hand, these two compounds were prepared easily in two steps.

**Scheme 5:**
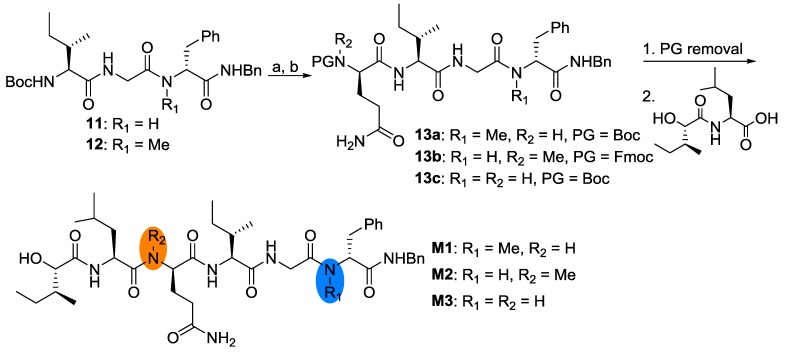
Reagents and conditions: (**a**) 4 mol/L HCl/EtOAc; (**b**) EDC/HOAt, Fmoc-Me-d-Gln-OH or Boc-d-Gln-OH. PG = protecting group.

Structurally, there are two *N*-methyl amino acids in the parent compound. Usually, *N*-methyl amino acids are believed to play important roles in the conformational alteration and increasing stability over proteolytic enzymes [9]. In order to investigate the role of the *N*-methyl amino acids, three analogues were designed (**M1**, **M2** and **M3**). Starting from easily obtained peptide fragments **11**, **12** and some commercially available reagents, these three compounds were obtained smoothly, as shown in Scheme 5.

### 2.2. Biological Results and Discussion

All these eighteen tasiamide derivatives prepared above were evaluated for cytotoxicity against KB and A549 cancer cell lines using etoposide as the positive control.

As shown in Table 1, none of the truncated analogues (**S1**–**S4**) were effective against KB or A549 cell lines even at 50 μM. This result indicated that a certain length of the peptide might be necessary for cytotoxicity. Analogues with full length, but some minor modifications at the *C*-terminus and/or glutamine residue (**C1**–**C8**) showed moderate activities against cancer cell lines. According to the IC_50_ values of eight compounds (**C1**–**C8**), l-Gln residue-containing analogues showed slightly better activity than their d-Gln counterparts (**C1** *vs.* **C2**, **C3** *vs.* **C4**, **C5** *vs.* **C6**, **C7** *vs.* **C8**). On the other hand, if Gln was replaced by Ala (**C9**), the cytotoxicity reduced dramatically. However, further structural optimizations of whether simplification on the Hmp residue (**N1**, **N2**) or modification on the *N*-methylated amino acids (**M1**–**M3**) led to inactive analogues.

**Table 1:** *In vitro* inhibitory activities of the tasiamide analogues (IC_50_ (µM) ^a^).

Compounds	KB ^b^	A549 ^b^	Compounds	KB ^b^	A549 ^b^
Etoposide ^c^	0.85 ± 0.08	0.99 ± 0.09	**C5**	2.45 ± 0.41	5.24 ± 1.12
**T2**	0.58 ± 0.07	>50	**C6**	8.52 ± 1.41	12.88 ± 1.32
**S1**	>50	>50	**C7**	3.80 ± 0.46	3.67 ± 0.66
**S2**	>50	>50	**C8**	3.64 ± 0.55	>50
**S3**	>50	>50	**C9**	>50	>50
**S4**	>50	>50	**N1**	>50	>50
**C1**	3.21 ± 0.6	4.17 ± 0.51	**N2**	>50	>50
**C2**	8.26 ± 1.22	>50	**M1**	>50	>50
**C3**	2.08 ± 0.33	2.24 ± 0.44	**M2**	>50	>50
**C4**	1.29 ± 0.31	8.48 ± 1.39	**M3**	>50	>50

^a^ The concentration of compound that inhibits 50% (IC_50_, µM) of the growth of human tumor cell line after 72 h of drug exposure. IC_50_ values are taken as the means ± standard deviation from three independent experiments; ^b^ KB, human nasopharyngeal carcinoma cell line; A549, human non-small cell lung tumor cell line; ^c^ Used as a positive control.

## 3. Experimental Section

### 3.1. Chemistry

#### 3.1.1. Materials and Methods

Solvents were processed by conventional methods. Thin layer chromatography (TLC) was performed on pre-coated Merck silica gel 60 F_254_ plates. Flash column chromatography was performed on silica gel (200–300 mesh, Qingdao Haiyang Chemical Co., Ltd, Qingdao, China). Optical rotations were determined with a JASCO P-1020 polarimeter. NMR spectra were recorded on a Jeol JNM-ECP 600 MHz spectrometer (Jeol Ltd., Tokyo, Japan) with Me_4_Si as the internal standard, and chemical shifts were recorded in *δ* value. Mass spectra were obtained on a Q-TOF GIOBAL mass spectrometer (Waters, Wilford, MA, USA).

#### 3.1.2. Synthesis of Boc-Ile-Gly-*N*-Me-d-Phe-Pro-OMe (**3**)

DEA (5 mL) was added to a solution of tripeptide **2** (428.4 mg, 0.75 mmol) in CH_3_CN (5 mL). The solution was concentrated *in vacuo* after stirring at room temperature (rt) for 2 h. Subsequently, the residue was then redissolved in CH_3_CN (5 mL), concentrated *in vacuo* again and dried under vacuum for 2 h. This free amine was dissolved in dry tetrahydrofuran (THF) (10 mL) and cooled with an ice-water bath for 10 min. Then, Boc-Ile-OH (208.0 mg, 0.9 mmol), EDC (173.0 mg, 0.9 mmol), HOAt (123.0 mg, 0.9 mmol) and NaHCO_3_ (130.0 mg, 1.5 mmol) were added, respectively. The mixture was stirred at 0 °C for 2 h and then rt overnight. The solvent was removed, and the residue was diluted with EtOAc (200 mL), washed with 10% citric acid, 5% NaHCO_3_ and brine. The organic layer was dried over Na_2_SO_4_, then concentrated *in vacuo*. The crude product was purified by flash chromatography providing Tetrapeptide (**3**) (306.8 mg, 73%): *R*_f_ (AcOEt/hexane 1:1) 0.41; 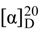 = 19.1 (*c* 0.05, CHCl_3_); ^1^H-NMR (CDCl_3_, 600 MHz) δ 7.31–7.08 (m, 5H), 6.82 (brs, 1H), 5.58 (t, *J* = 7.0 Hz, 1H), 5.03–4.99 (m, 1H), 4.42 (dd, *J* = 8.3, 5.5 Hz, 1H), 4.07–4.00 (m, 1H), 3.92–3.84 (m, 1H), 3.72 (s, 3H), 3.39–3.35 (m, 2H), 3.28 (dd, *J* = 13.7, 8.3 Hz, 1H), 2.99 (s, 3H), 2.84 (dd, *J* = 13.7, 7.1 Hz, 1H), 2.16–2.10 (m, 1H), 1.93–1.87 (m, 4H), 1.44–1.42 (m, 10H), 1.10–0.96 (m, 1H), 0.93–0.84 (m, 6H); ESI-MS *m/z*: 583.3 [M + Na]^+^; HRESIMS calcd. for C_29_H_44_N_4_O_7_Na [M + Na]^+^ 583.3108, found 583.3120.

#### 3.1.3. Synthesis of *N,N*-Me_2_-d-Gln-Ile-Gly-*N*-Me-d-Phe-Pro-OMe (**S1**)

A solution of compound **3** (91 mg, 0.162 mmol) in HCl/EtOAc (4 mol/L, 2 mL) was concentrated *in vacuo* after stirring for 45 min at rt. The residue was redissolved in EtOAc (5 mL) and concentrated *in vacuo* again. The resulting yellow solid was dried under vacuum for 2 h and then dissolved in dry THF (10 mL). After being cooled with an ice-water bath for 10 min, *N*,*N*-Me_2_-d-Gln(Trt)-OH (81.1 mg, 0.195 mmol), EDC (46.6 mg, 0.243 mmol), HOAt (33.1 mg, 0.243 mmol) and NaHCO_3_ (27.2 mg, 0.324 mmol) were added, respectively. The mixture was stirred at this temperature for 2 h and then at rt for another 12 h. The solvent was removed, and the residue was dissolved in EtOAc (100 mL) and washed with 10% citric acid, 5% NaHCO_3_ and brine. The organic layer was dried over Na_2_SO_4_ and then concentrated *in vacuo*. The residue was purified by flash column chromatography to give the desired pentapeptide **4** as a pale-yellow solid (133 mg, 95%).

125 mg (0.146 mmol) of compound **4** was dissolved in 5 mL of DCM and treated with TFA (5 mL) for 2 h, then concentrated *in vacuo*. The residue was redissolved in 5 mL of DCM and concentrated *in vacuo* again. The residue was purified by flash column chromatography to give the desired **S1** as a white solid (85 mg, 97%): *R*_f_ (CHCl_3_/MeOH 1:1) 0.30; 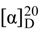 = 28.1 (*c* 0.14, CHCl_3_); ^1^H-NMR (CDCl_3_, 600 MHz) δ 9.30–9.10 (m, 1H), 8.79–8.71 (m, 1H), 8.00 (t, *J* = 7.8 Hz, 1H), 7.36–7.30 (m, 1H), 7.22–7.12 (m, 5H), 5.42 (t, *J* = 7.3 Hz, 1H), 4.51–4.49 (m, 1H), 4.35–4.31 (m, 2H), 4.10–3.99 (m, 1H), 3.82–3.79 (m, 1H), 3.70 (s, 3H), 3.26–3.24 (m, 1H), 3.23–3.19 (m, 1H), 3.00–2.95 (m, 4H), 2.85 (brs, 6H), 2.76–2.74 (m, 1H), 2.65–2.59 (m, 2H), 2.45–2.30 (m, 1H), 2.14–2.03 (m, 2H), 1.88–1.85 (m, 2H), 1.79–1.77 (m, 1H), 1.73–1.69 (m, 1H), 1.42–1.39 (m, 1H), 1.20–1.18 (m, 1H), 0.88–0.76 (m, 6H); ^13^C-NMR (CDCl_3_, 150 MHz) δ 172.6, 167.8, 136.9, 129.4, 128.5, 126.9, 59.5, 59.2, 59.1, 56.7, 52.4, 46.8, 41.2, 36.4, 35.1, 30.2, 29.8, 28.9, 25.2, 25.1, 24.9, 18.5, 15.6, 11.1; HRESIMS calcd. for C_31_H_48_N_6_O_7_Na [M + Na]^+^ 639.3482, found 639.3472.

#### 3.1.4. Synthesis of *N,N*-Me_2_-Ile-Gly-*N*-Me-d-Phe-Pro-OMe (**S2**)

DEA (5 mL) was added to a solution of tripeptide **2** (62.5 mg, 0.11 mmol) in CH_3_CN (5 mL). After the solution was stirred at rt for 2 h, the solvent was concentrated *in vacuo*. The residue was redissolved in CH_3_CN (5 mL), concentrated *in vacuo* and dried under vacuum for 2 h. This free amine was dissolved in dry DCM (10 mL) and cooled with an ice-water bath for 10 min. Then, *N*, *N*-Me_2_-Ile-OH (35.0 mg, 0.22 mmol), EDC (31.6 mg, 0.17 mmol), HOAt (22.5 mg, 0.17 mmol), and DIEA (38.4 μL, 0.122 mmol) were added, respectively. The mixture was stirred at this temperature for 2 h and then at rt overnight. The reaction mixture was diluted with 80 mL of EtOAc and washed with 10% citric acid, 5% NaHCO_3_ and brine. The organic layer was dried over Na_2_SO_4_ and then concentrated *in vacuo*. The residue was purified by flash column chromatography to give the desired compound **S2** as a pale-yellow solid (13.0 mg, 24%): *R*_f_ (CHCl_3_/MeOH 10:1) 0.72; 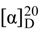 = 26.5 (*c* 0.13, CHCl_3_); ^1^H-NMR (CDCl_3_, 600 MHz) δ 7.32–7.19 (m, 5H), 6.80–6.78 (m, 1H), 5.50 (t, *J* = 7.8 Hz, 1H), 4.50–4.44 (m, 1H), 4.41 (m, 1H), 4.10 (d, *J* = 4.1 Hz, 1H), 3.90 (d, *J* = 3.2 Hz, 1H), 3.70 (s, 3H), 3.37 (t, *J* = 4.6 Hz, 2H), 3.28 (dd, *J* = 13.7, 8.2 Hz, 1H), 3.00 (s, 3H), 2.85 (dd, *J* = 13.7, 7.3 Hz, 1H), 2.26–2.00 (brs, 6H), 1.97–1.81 (m, 4H), 1.72–1.70 (m, 1H), 1.55–1.42 (m, 2H), 0.99–0.81 (m, 6H); ESI-MS *m/z*: 511.2 [M + Na]^+^; HRESIMS calcd. for C_26_H_40_N_4_O_5_Na [M + Na]^+^ 511.2896, found 511.2903.

#### 3.1.5. Synthesis of HO-Hmp-Leu-*N*-Me-d-Gln-Ile-OBn (**S3**)

Hydrogenation of the benzyl ester, **6** (69.4 mg, 0.21 mmol), was carried out in EtOAc (10 mL) in the presence of a catalytic amount of Pd–C (10%) under hydrogen for 4 h. The Pd–C was removed by filtration, and the filtrate was concentrated *in vacuo* to yield the corresponding carboxylic acid, **6a**, which was used for the next step without further purification.

DEA (5 mL) was added to a solution of dipeptide **5** (111.3 mg, 0.19 mmol) in CH_3_CN (5 mL). After the solution was stirred at rt for 2 h, the solvent was concentrated *in vacuo*. The residue was redissolved in CH_3_CN (5 mL), concentrated *in vacuo* again, then dried under vacuum for 2 h. This free amine (**5a**) was dissolved in DCM (10 mL) and cooled with an ice-water bath. EDC (54.1 mg 0.28 mmol), HOAt (38.4 mg, 0.28 mmol) and NaHCO_3_ (23.7 mg, 0.28 mmol) were added, respectively. The mixture was stirred at this temperature for 2 h and then at rt overnight. The reaction mixture was diluted with 100 mL of EtOAc and washed with 10% citric acid, 5% NaHCO_3_ and brine. The organic layer was dried over Na_2_SO_4_ and then concentrated *in vacuo*. The residue was purified by flash column chromatography to give the desired compound **S3** as a white solid (94.4 mg, 85%): *R*_f_ (CHCl_3_/MeOH 15:1) 0.30; 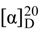 = −48.5 (*c* 0.29, CHCl_3_); ^1^H-NMR (CDCl_3_, 600 MHz) δ 7.35–7.28 (m, 5H), 7.20–7.18 (m, 1H), 7.12–7.09 (m, 1H), 6.94 (brs, 1H), 6.64 (brs, 1H), 5.20 (d, *J* = 12.1 Hz, 1H), 5.10 (d, *J* = 12.1 Hz, 1H), 4.76–4.73 (m, 1H), 4.50–4.47 (m, 1H), 3.95–3.91 (m, 1H), 3.11–2.82 (m, 4H), 2.30–2.12 (m, 3H), 2.05–1.83 (m, 3H), 1.71–1.65 (m, 3H), 1.44–1.11 (m, 4H), 0.98–0.73 (m, 18H); ^13^C-NMR (CDCl_3_, 150 MHz) δ 175.9, 175.2, 174.5, 171.9, 170.4, 135.4, 128.7, 128.6, 128.5, 76.3, 67.2, 56.8, 54.6, 48.1, 40.7, 40.1, 38.6, 37.4, 32.0, 30.7, 29.8, 25.0, 23.7, 23.4, 21.3, 15.7, 14.2, 11.9, 11.6; HRESIMS calcd. for C_31_H_50_N_4_O_7_Na [M + Na]^+^ 613.3577, found 613.3589.

#### 3.1.6. Synthesis of HO-Hmp-Leu-*N*-Me-d-Gln-Ile-Gly-OBn (**S4**)

Hydrogenation of **S3** (23.6 mg, 0.04 mmol) was carried out in EtOAc-EtOH (1:4, 10 mL) in the presence of a catalytic amount of Pd–C (10%) under hydrogen for 4 h. The Pd–C was removed by filtration, and the filtrate was concentrated *in vacuo* to yield the corresponding carboxylic acid **S3a**, which was used for next step without further purification.

H-Gly-OBn tosylate (16.4 mg, 0.05 mmol) and the carboxylic acid obtained above were dissolved in dry THF and cooled with an ice-water bath for 15 min. EDC (9.4 mg, 0.05 mmol), HOAt (6.7 mg, 0.05 mmol) and NMM (5.4 μL, 0.05 mmol) were added, respectively. The mixture was stirred at this temperature for 2 h and then at rt overnight. The reaction mixture was diluted with 50 mL of EtOAc and washed with 10% citric acid, 5% NaHCO_3_ and brine. The organic layer was dried over Na_2_SO_4_ and then concentrated *in vacuo*. The residue was purified by flash column chromatography to give the desired compound **S4** as a white solid (15.6 mg, 60%): *R*_f_ (AcOEt/hexane 1:1) 0.25; 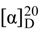 = −37 (*c* 0.07, MeOH); ^1^H-NMR (CDCl_3_, 600 MHz) δ 7.37–7.32 (m, 5H), 7.25 (d, *J* = 7.7 Hz, 1H), 7.09 (d, *J* = 8.8 Hz, 1H), 6.25 (brs, 1H), 5.98 (brs, 1H), 5.15 (d, *J* = 13.3 Hz, 2H), 5.15 (t, *J* = 7.7 Hz, 1H), 4.84–4.78 (m, 1H), 4.53 (brs, 1H), 4.32 (t, *J* = 7.7 Hz, 1H), 4.07 (m, 1H), 3.94–3.93 (m, 1H), 3.92 (d, *J* = 4.7 Hz, 1H), 3.08 (s, 3H), 2.34–2.27 (m, 1H), 2.26–2.20 (m, 1H), 2.19–2.14 (m, 1H), 2.01–1.95 (m, 1H), 1.87–1.82 (m, 1H), 1.80–1.77 (m, 1H), 1.68–1.60 (m, 2H), 1.58–1.52 (m, 1H), 1.51–1.45 (m, 1H), 1.44–1.37 (m, 1H), 1.24–1.19 (m, 1H), 1.12–1.06 (m, 1H), 0.97 (d, *J* = 6.6 Hz, 3H), 0.94 (d, *J* = 7.0 Hz, 3H), 0.90–0.83 (m, 9H); ^13^C-NMR (CDCl_3_, 150 MHz) δ: 174.7, 174.6, 172.0, 169.9, 169.5, 135.0, 128.6, 128.5, 128.4, 76.3, 67.2, 58.2, 56.4, 47.7, 41.3, 40.6, 38.4, 36.8, 32.3, 31.3, 25.0, 24.9, 23.7, 23.2, 23.1, 21.9, 15.6, 15.4, 11.8, 11.2; ESI-MS *m/z*: 670.4 [M + Na]^+^; HRESIMS calcd. for C_33_H_53_N_5_O_8_Na [M + Na]^+^ 670.3792, found 670.3808.

#### 3.1.7. General Procedure for the Preparation of Compounds **7a**–**d**

To a solution of Boc-*N*-Me-d-Phe-OH (1.0 mmol) and amine (1.1 mmol of aniline, phenylmethanamine, tetrahydroisoquinoline or tetrahydroisoquinoline carboxylate) in DCM-DMF (1:1, 10 mL) at 0 °C were added EDC (1.2 mmol), HOAt (1.2 mmol) and NaHCO_3_ (1.2 mmol), respectively. The mixture was stirred at this temperature for 2 h and then at rt overnight. The reaction mixture was diluted with 100 mL of EtOAc and washed with 10% citric acid, 5% NaHCO_3_ and brine. The organic layer was dried over Na_2_SO_4_ and then concentrated *in vacuo*. The residue was purified by flash column chromatography to give the desired compounds.

Boc-*N*-Me-d-Phe-NH-Ph (**7a**): Yield 65%; *R*_f_ (AcOEt/hexane 1:1) 0.80; ^1^H-NMR (CDCl_3_, 600 MHz) δ 8.31 (brs, 1H), 7.60–7.09 (m, 10H), 5.00 (brm, 1H), 3.54–3.37 (m, 1H), 3.08–3.01 (m, 1H), 2.81 (s, 3H), 1.43(s, 9H, Boc); ESI-MS *m/z*: 377.2 [M + Na]^+^; HRESIMS calcd. for C_21_H_26_N_2_O_3_Na [M + Na]^+^ 377.1841, found 377.1853.

Boc-*N*-Me-d-Phe-NH-Bn (**7b**): Yield 57%; *R*_f_ (AcOEt/hexane 1:1) 0.81; ^1^H-NMR (CDCl_3_, 600 MHz) δ 7.34–7.15 (m, 10H), 6.50 (brs, 1H), 4.92 (t, *J* = 7.8 Hz, 1H), 4.51 (dd, *J* = 14.6, 6.4 Hz, 1H), 4.43 (dd, *J* = 14.6, 5.0 Hz, 1H), 3.51–3.36 (m, 1H), 3.00–2.89 (m, 1H), 2.76 (s, 3H), 1.35 (s, 9H); ESI-MS *m/z*: 391.3 [M + Na]^+^; HRESIMS calcd. for C_22_H_28_N_2_O_3_Na [M + Na]^+^ 391.1998, found 391.2006.

Boc-*N*-Me-d-Phe-NH-MTC (**7c**, MTC = methyl (*S*)-1,2,3,4-tetrahydroisoquinoline-3-carboxylate): Yield 95%; *R*_f_ (AcOEt/hexane 1:1) 0.78; ^1^H-NMR (CDCl_3_, 600 MHz) δ 7.29–7.12 (m, 9H), 5.45 (dd, *J* = 5.0, 4.5 Hz, 1H), 5.39 (dd, *J* = 7.8, 7.3 Hz, 1H), 4.52–4.49 (m, 1H), 4.35–4.32 (m, 1H), 3.65 (s, 3H), 3.60–3.18 (m, 2H), 3.15–3.02 (m, 2H), 2.96 (s, 3H), 1.38 (s, 9H); ESI-MS *m/z*: 475.2 [M + Na]^+^; HRESIMS calcd. for C_26_H_32_N_2_O_5_Na [M + Na]^+^ 475.2209, found 475.2231.

Boc-*N*-Me-d-Phe-NH-TIQ (**7d**, TIQ = 1,2,3,4-tetrahydroisoquinoline): Yield 63%; *R*_f_ (AcOEt/hexane 1:1) 0.68; ^1^H-NMR (CDCl_3_, 600 MHz) δ 7.30–7.08 (m, 9H), 5.38–5.36 (m, 1H), 5.05–4.49 (m, 2H), 4.11–3.19 (m, 2H), 3.22–2.96 (m, 2H), 2.85–2.81 (m, 1H), 2.76–2.65 (m, 1H), 2.79 (s, 3H), 1.35 (s, 9H); ESI-MS *m/z*: 417.2 [M + Na]^+^; HRESIMS calcd. for C_24_H_30_N_2_O_3_Na [M + Na]^+^ 417.2154, found 417.2161.

#### 3.1.8. General Procedure for the Preparation of Compounds **8a**–**d**

Compound **7a** (or **7b**–**d**) (0.13 mmol) was treated with HCl/EtOAc (4 mol/L, 2 mL) for 45 min then concentrated *in vacuo*. The residue was redissolved in EtOAc (5 mL) and concentrated *in vacuo* again. The resulting solid was dried under vacuum for 2 h and then dissolved in 4 mL of dry DCM-DMF (3:1). After being cooled with an ice-water bath for 10 min, Fmoc-Gly-OH (0.13 mmol), EDC (0.16 mmol), HOAt (0.16 mmol) and NaHCO_3_ (0.16 mmol) were added, respectively. The mixture was stirred at this temperature for 2 h and then at rt for another 12 h. The mixture was diluted with 80 mL of EtOAc and washed with 10% citric acid, 5% NaHCO_3_, water and brine. The organic layer was dried over Na_2_SO_4_ and then concentrated *in vacuo*. The residue was purified by flash column chromatography to give the desired compound **8a** (or **8b**–**d**).

Fmoc-Gly-*N*-Me-d-Phe-NH-Ph (**8a**): Yield 64%; *R*_f_ (AcOEt/hexane 1:1) 0.41; ^1^H-NMR (CDCl_3_, 600 MHz) δ 7.76 (d, *J* = 7.8 Hz, 2H), 7.60 (d, *J* = 5.9 Hz, 2H), 7.47 (d, *J* = 7.8 Hz, 1H), 7.40 (t, *J* = 7.3 Hz, 2H), 7.31 (t-like, *J* = 7.3, 6.4 Hz, 2H), 7.29–7.22 (m, 10H), 5.67 (brm, 1H), 5.41 (t, *J* = 7.8 Hz, 1H), 4.38 (d, *J* = 7.3 Hz, 2H), 4.21 (t-like, *J* = 7.3, 6.8 Hz, 1H), 4.04 (dd, *J* = 17.0, 4.6 Hz, 1H), 3.89 (dd, *J* = 17.0, 4.6 Hz, 1H), 3.42 (dd, *J* = 14.2, 7.3 Hz, 1H), 3.06 (dd, *J* = 14.2, 8.3 Hz, 1H) , 3.00 (s, 3H); ESI-MS *m/z*: 556.2 [M + Na]^+^; HRESIMS calcd. for C_33_H_31_N_3_O_4_Na [M + Na]^+^ 556.2212, found 556.2231.

Fmoc-Gly-*N*-Me-d-Phe-NH-Bn (**8b**): Yield 99%; *R*_f_ (AcOEt/hexane 1:1) 0.43; ^1^H-NMR (CDCl_3_, 600 MHz) δ 7.76 (d, *J* = 7.8 Hz, 2H), 7.59 (d, *J* = 7.3 Hz, 2H), 7.40 (t, *J* = 7.3 Hz, 2H), 7.31 (t, *J* = 7.3 Hz, 2H), 7.30–7.09 (m, 10H), 6.31 (br, 1H), 5.63 (br, 1H), 5.31 (t-like, *J* = 8.2, 7.8 Hz, 1H), 4.44–4.42 (m, 1H), 4.36 (d, *J* = 6.8 Hz, 2H), 4.31–4.29 (m, 1H), 4.21 (t, *J* = 7.3 Hz, 1H), 3.99 (dd, *J* = 16.9, 4.6 Hz, 1H), 3.84 (dd, *J* = 17.4, 4.6 Hz, 1H), 3.37 (dd, *J* = 13.7, 7.8 Hz, 1H), 3.00 (dd, *J* = 14.2, 8.3 Hz, 1H) , 2.94 (s, 3H); ESI-MS *m/z*: 570.2 [M + Na]^+^; HRESIMS calcd. for C_34_H_33_N_3_O_4_Na [M + Na]^+^ 570.2369, found 570.2381.

Fmoc-Gly-*N*-Me-d-Phe-NH-MTC (**8c**): Yield 53%; *R*_f_ (AcOEt/hexane 1:1) 0.38; ^1^H-NMR (CDCl_3_, 600 MHz) δ 7.78 (d, *J* = 7.8 Hz, 2H), 7.62 (m, 2H), 7.41 (t-like, *J* = 7.3, 7.8 Hz, 2H), 7.33 (t, *J* = 7.3 Hz, 2H), 7.26–7.06 (m, 9H), 5.69–5.67 (m, 1H), 4.60 (d, *J* = 15.6 Hz, 1H), 4.39–4.37 (m, 2H), 4.24–4.21 (m, 1H), 4.12 (dd, *J* = 14.6, 7.3 Hz, 1H), 4.11–4.08 (m, 1H), 3.88–3.85 (m, 1H), 3.79–3.77 (m, 1H), 3.65 (s, 3H), 3.30–3.26 (m, 1H), 3.11 (dd, *J* = 15.5, 5.0 Hz, 1H), 3.09 (dd, *J* = 16.5, 6.4 Hz, 1H), 3.00 (s, 3H); 2.98–2.94 (m, 1H); ESI-MS *m/z*: 632.3 [M + H]^+^, 654.3 [M + Na]^+^; HRESIMS calcd. for C_38_H_37_N_3_O_6_Na [M + Na]^+^ 654.2580, found 654.2598.

Fmoc-Gly-*N*-Me-d-Phe-NH-TIQ (**8d**): Yield 97%; *R*_f_ (AcOEt/hexane 1:1) 0.34; ^1^H-NMR (CDCl_3_, 600 MHz) δ 7.77 (dd, *J* = 7.3, 3.2 Hz, 2H), 7.61 (t, *J* = 6.4 Hz, 2H), 7.41 (m, 2H), 7.32 (dd, *J* = 13.0, 7.0 Hz, 2H), 7.24–7.05 (m, 9H), 6.93 (d, *J* = 7.8 Hz, 1H), 5.65 (m, 1H), 4.76 (d, *J* = 16.5 Hz, 1H), 4.38–4.35 (m, 2H), 4.22 (dd, *J* = 12.8, 6.8 Hz, 1H), 4.12 (dd, *J* = 14.2, 6.8 Hz, 1H), 4.03–3.98 (m, 1H), 3.90–3.86 (dd, *J* = 16.9, 4.1 Hz, 1H), 3.69–3.64 (m, 1H), 3.64–3.54 (m, 1H), 3.32–3.24 (m, 1H), 3.01 (s, 3H), 2.98–2.88 (m, 1H), 2.86–2.84 (m, 1H), 2.79–2.55 (m, 1H); ESI-MS *m/z*: 574.3 [M + H]^+^, 596.3 [M + Na]^+^; HRESIMS calcd. for C_36_H_35_N_3_O_4_Na [M + Na]^+^ 596.2525, found 596.2547.

#### 3.1.9. General Procedure for the Preparation of Compounds **C1**–**C9**

Following the similar procedure described previously [5]:

HO-Hmp-Leu-*N*-Me-Gln-Ile-Gly-*N*-Me-d-Phe-NH-Ph (**C1**): Yield 75%; *R*_f_ (CHCl_3_/MeOH 20:1) 0.21; 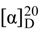 = −17.0 (*c* 0.01 MeOH); ^1^H-NMR (CDCl_3_, 600 MHz) δ 7.83 (d, *J* = 7.7 Hz, 1H), 7.68 (d, *J* = 7.7 Hz, 1H), 7.51–7.06 (m, 11H), 5.34 (t, *J* = 7.7 Hz, 1H), 5.07 (dd, *J* = 10.4, 3.3 Hz, 1H), 4.80 (brs, 1H), 4.69–4.66 (m, 1H), 4.42 (dd, *J* = 8.1, 3.7 Hz, 1H), 4.12–4.07 (m, 1H), 3.93 (dd, *J* = 13.2, 4.0 Hz, 1H), 3.88 (d, *J* = 3.3 Hz, 1H), 3.43 (dd, *J* = 13.9, 8.0 Hz, 1H), 3.15 (s, 3H), 2.99 (s, 3H), 2.99–2.93 (m, 1H), 2.33–2.15 (m, 3H), 2.10–1.93 (m, 3H), 1.83–1.67 (m, 3H), 1.46–1.39 (m, 1H), 1.26–1.09 (m, 3H), 0.96–0.79 (m, 18H); ^13^C-NMR (CDCl_3_, 150 MHz) δ 177.5, 173.2, 171.3, 170.4, 169.6, 167.3, 138.5, 137.4, 129.1, 129.0, 128.8, 128.7, 126.9, 124.1, 120.2, 119.7, 63.8, 57.9, 54.1, 49.7, 41.4, 38.6, 38.5, 38.0, 36.7, 36.4, 34.4, 31.9, 31.1, 30.4, 30.3, 30.2, 30.0, 29.9, 29.7, 29.4, 28.8, 27.1, 25.1, 24.5, 24.1, 23.9, 23.8, 23.7, 23.5, 23.4, 22.7, 20.9, 20.5, 15.7, 15.6, 15.4, 15.3, 14.1, 12.2, 12.0, 11.9, 11.5; ESI-MS *m/z*: 794.4 [M + H]^+^, 816.4 [M + Na]^+^; HRESIMS calcd. for C_42_H_63_N_7_O_8_Na [M + Na]^+^ 816.4636, found 816.4668.

HO-Hmp-Leu-*N*-Me-d-Gln-Ile-Gly-*N*-Me-d-Phe-NH-Ph (**C2**): Yield 67%; *R*_f_ (CHCl_3_/MeOH 20:1) 0.23; 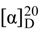 = −27.5 (*c* 0.02 MeOH); ^1^H-NMR (CDCl_3_, 600 MHz) δ 7.30–7.06 (m, 13H), 6.15 (brs, 1H), 5.89 (brs, 1H), 5.28–5.22 (m, 1H), 5.05 (t, *J* = 9.1 Hz, 1H), 4.95–4.90 (m, 1H), 4.82 (d, *J* = 4.8 Hz, 1H), 4.31–4.29 (m, 1H), 4.04–4.00 (m, 1H), 3.91 (d, *J* = 3.7 Hz, 1H), 3.78–3.73 (m, 1H), 3.37 (dd, *J* = 17.4, 6.9 Hz, 1H), 3.08–2.92 (m, 4H), 2.82 (s, 3H), 2.39 (brs, 1H), 2.33–2.28 (m, 1H), 2.23–2.14 (m, 1H), 1.99–1.97 (m, 1H), 1.84–1.82 (m, 2H), 1.64–1.57 (m, 3H), 1.45–1.41 (m, 2H), 1.24–1.17 (m, 1H), 1.13–1.04 (m, 1H), 0.99–0.93 (m, 9H), 0.89–0.84 (m, 9H); ^13^C-NMR (CDCl_3_, 150 MHz) δ 174.4, 174.3, 174.0, 171.4, 170.3, 169.7, 168.2, 136.5, 128.7, 128.6, 127.0, 76.5, 58.8, 57.8, 47.2, 41.3, 41.1, 40.6, 38.5, 38.4, 38.3, 37.3, 34.7, 32.2, 31.6, 31.1, 29.7, 25.0, 24.8, 23.7, 23.1, 23.0, 22.9, 22.1, 21.7, 15.6, 14.2, 11.8, 11.3; ESI-MS *m/z*: 794.4 [M + H]^+^; HRESIMS calcd. for C_42_H_63_N_7_O_8_Na [M + Na]^+^ 816.4636, found 816.4670.

HO-Hmp-Leu-*N*-Me-Gln-Ile-Gly-*N*-Me-d-Phe-NH-Bn (**C3**): Yield 63%; *R*_f_ (CHCl_3_/MeOH 20:1) 0.28; 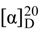 = −19.7 (*c* 0.01 MeOH); ^1^H-NMR (CDCl_3_, 600 MHz) δ 7.99 (brs, 1H), 7.73 (brs, 1H), 7.54 (d, *J* = 8.5 Hz, 1H),7.27–7.16 (m, 5H), 7.11 (d, *J* = 7.0 Hz, 2H), 5.31 (t, *J* = 7.7 Hz, 1H), 5.13 (dd, *J* = 10.3, 4.4 Hz, 1H), 4.76–4.71 (m, 1H), 4.45 (dd, *J* = 14.6, 6.6 Hz, 1H), 4.31 (dd, *J* = 7.7, 5.5 Hz, 1H), 4.25 (dd, *J* = 9.0, 5.4 Hz, 1H), 3.94–3.80 (m, 3H), 3.47 (s, 3H), 3.38 (dd, *J* = 14.3, 8.0 Hz, 1H), 2.96 (s, 3H), 2.96–2.86 (m, 1H), 2.33–1.94 (m, 4H), 1.88–1.92 (m, 1H), 1.77–1.70 (m, 2H), 1.45–1.29 (m, 3H), 1.21–1.15 (m, 2H), 1.08–1.01 (m, 1H), 0.98–0.80 (m, 18H); ^13^C-NMR (CDCl_3_, 150 MHz) δ 176.8, 176.0, 175.7, 174.7, 171.3, 170.7, 170.3, 169.2, 138.4, 138.2, 137.5, 136.8, 129.2, 129.0, 128.9, 128.6, 128.5, 127.7, 127.5, 127.3, 127.2, 126.8, 76.6, 76.5, 60.4, 58.1, 58.0, 50.8, 49.0, 48.2, 48.1, 43.6, 43.3, 41.3, 39.5, 38.5, 38.4, 36.6, 34.5, 34.4, 31.3, 30.5, 25.0, 24.9, 24.8, 24.6, 24.0, 23.9, 23.5, 23.4, 21.6, 21.1, 20.8, 15.7, 15.5, 15.4, 14.2, 11.9, 11.8, 11.4; ESI-MS *m/z*: 808.4 [M + H]^+^; HRESIMS calcd. for C_43_H_65_N_7_O_8_Na [M + Na]^+^ 830.4792, found 830.4814.

HO-Hmp-Leu-*N*-Me-d-Gln-Ile-Gly-*N*-Me-d-Phe-NH-Bn (**C4**): Yield 77%; *R*_f_ (CHCl_3_/MeOH 20:1) 0.22; 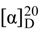 = −7.3 (*c* 0.05 MeOH); ^1^H-NMR (CDCl_3_, 600 MHz) δ 7.27–7.06 (m, 12H), 5.95 (brs, 1H), 5.69 (brs, 1H), 5.31 (t, *J* = 8.0 Hz, 1H), 4.96 (t, *J* = 7.3 Hz, 1H), 4.94–4.91 (m, 1H), 4.43 (dd, *J* = 14.9, 6.0 Hz, 1H), 4.37 (dd, *J* = 13.7, 6.1 Hz, 1H), 4.26 (dd, *J* = 14.8, 5.3 Hz, 1H), 4.03 (dd, *J* = 17.6, 4.7 Hz, 1H), 3.89 (d, *J* = 4.2 Hz, 1H), 3.78 (d, *J* = 17.2 Hz, 1H), 3.33 (dd, *J* = 14.1, 8.3 Hz, 1H), 3.00 (s, 3H), 2.99–2.92 (m, 1H), 2.96 (s, 3H), 2.25–2.19 (m, 1H), 2.18–2.07 (m, 2H), 1.97–1.91 (m, 1H), 1.86–1.84 (m, 1H), 1.80–1.76 (m, 1H), 1.64–1.58 (m, 1H), 1.58–1.55 (m, 1H), 1.47–1.39 (m, 3H), 1.21–1.14 (m, 1H), 1.12–1.04 (m, 1H), 0.95–0.93 (m, 6H), 0.92 (d, *J* = 7.0 Hz, 3H), 0.89–0.84 (m, 9H); ^13^C-NMR (CDCl_3_, 150 MHz) δ 175.1, 174.7, 174.5, 174.3, 174.1, 171.6, 171.5, 169.9, 169.4, 168.8, 168.3, 168.2, 138.3, 138.0, 137.1, 136.7, 129.1, 128.9, 128.5, 127.6, 127.5, 127.3, 127.2, 127.1, 126.8, 77.1, 62.0, 58.0, 57.9, 57.7, 56.7, 53.4, 47.4, 47.2, 43.5, 43.3, 41.3, 41.2, 40.4, 38.5, 38.4, 36.9, 36.1, 35.1, 34.8, 32.0, 31.9, 31.3, 30.1, 30.4, 29.7, 29.4, 24.9, 24.7, 24.2, 23.8, 23.2, 23.1, 22.7, 22.0, 21.7, 15.7, 15.5, 15.2, 14.1, 11.8, 11.7, 11.3, 11.0; ESI-MS *m/z*: 808.5 [M + H]^+^, 830.5 [M + Na]^+^; HRESIMS calcd. for C_43_H_65_N_7_O_8_Na [M + Na]^+^ 830.4792, found 830.4828.

HO-Hmp-Leu-*N*-Me-Gln-Ile-Gly-*N*-Me-d-Phe-NH-MTC (**C5**): Yield 60%; *R*_f_ (CHCl_3_/MeOH 20:1) 0.29; 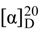 = −9.9 (*c* 0.05 MeOH); ^1^H-NMR (CDCl_3_, 600 MHz) δ 7.36 (d, *J* = 8.0 Hz, 1H), 7.31 (d, *J* = 7.7 Hz, 1H), 7.28–6.99 (m, 9H), 6.96 (t, *J* = 4.4 Hz, 1H), 6.73 (brs, 1H), 6.18 (brs, 1H), 5.78 (t, *J* = 7.5 Hz, 1H), 5.21 (t, *J* = 5.5 Hz, 1H), 5.13 (t, *J* = 7.7 Hz, 1H), 4.85–4.81 (m, 1H), 4.53 (d, *J* = 15.8 Hz, 1H), 4.43 (d, *J* = 15.4 Hz, 1H), 4.29 (t, *J* = 7.3 Hz, 1H), 4.17 (dd, *J* = 17.4, 5.0 Hz, 1H), 3.98 (brs, 1H), 3.84 (dd, *J* = 17.6, 3.7 Hz, 1H), 3.64 (s, 3H), 3.27 (dd, *J* = 13.7, 7.5 Hz, 1H), 3.21–3.15 (m, 1H), 3.14 (s, 3H), 3.11–3.04 (m, 1H), 3.01 (s, 3H), 2.93 (dd, *J* = 13.7, 7.5 Hz, 1H), 2.36–2.32 (m, 1H), 2.23–2.18 (m, 2H), 2.07–2.02 (m, 1H), 1.96–1.93 (m, 1H), 1.86–1.82 (m, 1H), 1.73–1.68 (m, 1H), 1.66–1.61 (m, 1H), 1.47–1.38 (m, 3H), 1.25–1.20 (m, 1H), 1.16–1.09 (m, 1H), 0.99–0.94 (m, 9H), 0.91–0.85 (m, 9H); ^13^C-NMR (CDCl_3_, 150 MHz) δ 175.0, 174.1, 171.1, 170.5, 169.2, 168.1, 136.7, 132.2, 132.1, 129.3, 128.5, 128.1, 127.5, 127.1, 126.9, 126.1, 76.2, 58.0, 55.4, 55.2, 52.6, 52.4, 47.9, 45.1, 41.2, 40.5, 38.5, 36.6, 35.2, 31.5, 30.7, 30.5, 29.7, 24.9, 24.7, 23.9, 23.6, 23.4, 21.3, 15.7, 15.6, 11.9, 11.3; ESI-MS *m/z*: 892.4 [M + H]^+^, 914.4 [M + Na]^+^; HRESIMS calcd. for C_47_H_69_N_7_O_10_Na [M + Na]^+^ 914.5004, found 914.5018.

HO-Hmp-Leu-*N*-Me-d-Gln-Ile-Gly-*N*-Me-d-Phe-NH-MTC (**C6**): Yield 67%; *R*_f_ (CHCl_3_/MeOH 20:1); 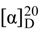 = −29.1 (*c* 0.01 MeOH); ^1^H-NMR (CDCl_3_, 600 MHz) δ 7.29–6.97 (m, 12H), 5.81 (t, *J* = 7.7 Hz, 2H), 5.57 (brs, 1H), 5.20 (dd, *J* = 6.2, 4.8 Hz, 1H), 5.07 (t, *J* = 7.5 Hz, 1H), 4.98 (dd, *J* = 15.4, 6.9 Hz, 1H), 4.53 (d, *J* = 15.4 Hz, 1H), 4.45 (d, *J* = 15.4 Hz, 1H), 4.33 (dd, *J* = 9.2, 6.6 Hz, 1H), 4.10 (dd, *J* = 17.2, 4.7 Hz, 1H), 3.93 (dd, *J* = 4.2 Hz, 1H), 3.80 (dd, *J* = 17.4, 3.5 Hz, 1H), 3.64 (s, 3H), 3.28 (dd, *J* = 13.9, 7.7 Hz, 1H), 3.17 (dd, *J* = 15.8, 6.4 Hz, 1H), 3.06 (dd, *J* = 15.8, 6.2 Hz, 1H), 3.02 (s, 3H), 3.00 (s, 3H), 2.92 (dd, *J* = 13.7, 7.5 Hz, 1H), 2.33 (td, *J* = 13.9, 7.4 Hz, 1H), 2.28–2.22 (m, 1H), 2.15–2.12 (m, 1H), 2.05–1.97 (m, 1H), 1.95 (brs, 1H), 1.88–1.80 (m, 1H), 1.65–1.60 (m, 2H), 1.51–1.39 (m, 3H), 1.23–1.19 (m, 1H), 1.17–1.10 (m, 1H), 0.97–0.95 (m, 9H), 0.93–0.86 (m, 9H); ESI-MS *m/z*: 892.4 [M + H]^+^; HRESIMS calcd. For C_47_H_69_N_7_O_10_Na [M + Na]^+^ 914.5004, found 914.5032.

HO-Hmp-Leu-*N*-Me-Gln-Ile-Gly-*N*-Me-d-Phe-NH-TIQ (**C7**): Yield 60%; *R*_f_ (CHCl_3_/MeOH 20:1) 0.28; 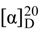 = −3.3 (*c* 0.02 MeOH); ^1^H-NMR (CDCl_3_, 600 MHz) δ 7.57 (d, *J* = 8.8 Hz, 1H), 7.40 (d, *J* = 8.8 Hz, 1H), 7.24–7.05 (m, 9H), 7.00 (t, *J* = 4.2 Hz, 1H), 6.80 (brs, 1H), 6.53 (brs, 1H), 5.76 (t, *J* = 7.5 Hz, 1H), 5.17 (dd, *J* = 9.8, 5.9 Hz, 1H), 4.81–4.76 (m, 1H), 4.72 (d, *J* = 16.5 Hz, 1H), 4.58 (d, *J* = 16.5 Hz, 1H), 4.26 (t, *J* = 7.3 Hz, 1H), 4.06 (dd, *J* = 17.6, 4.7 Hz, 1H), 4.00 (dt, *J* = 12.8, 5.9 Hz, 1H), 3.96 (d, *J* = 2.9 Hz, 1H), 3.88 (dd, *J* = 17.6, 3.7 Hz, 1H), 3.72 (t, *J* = 3.8 Hz, 1H), 3.64 (dd, *J* = 7.3, 4.7 Hz, 1H), 3.57 (dd, *J* = 8.4, 4.7 Hz, 1H), 3.51 (dd, *J* = 7.7, 4.7 Hz, 1H), 3.30 (dd, *J* = 13.6, 8.0 Hz, 1H), 3.15 (s, 3H), 2.94–2.88 (m, 1H), 2.86–2.82 (m, 1H), 2.79 (s, 3H), 2.79–2.76 (m, 1H), 2.26–2.19 (m, 1H), 2.18–2.13 (m, 2H), 2.08–2.01 (m, 1H), 1.93–1.88 (m, 1H), 1.84–1.81 (m, 1H), 1.75–1.70 (m, 1H), 1.69–1.64 (m, 1H), 1.45–1.38 (m, 3H), 1.25–1.19 (m, 1H), 1.12–1.07 (m, 1H), 0.98–0.93 (m, 9H), 0.83–0.91 (m, 9H); ^13^C-NMR (CDCl_3_, 150 MHz) δ 175.5, 175.4, 174.2, 171.2, 171.0, 170.6, 168.6, 168.2, 167.9, 136.7, 134.2, 134.0, 132.8, 132.7, 129.2, 128.6, 128.5, 128.4, 127.1, 126.9, 126.6, 126.5, 126.4, 76.4, 57.9, 54.8, 54.7, 54.0, 48.0, 47.1, 44.7, 43.1, 41.1, 41.3, 41.2, 40.1, 40.0, 38.5, 36.6, 36.5, 35.7, .35.4, 31.4, 30.6, 30.0, 29.7, 29.5, 29.3, 24.9, 24.7, 24.0, 23.7, 23.4, 21.2, 15.6, 15.5, 11.8, 11.3; ESI-MS *m/z*: 834.3 [M + H]^+^, 856.3 [M + Na]^+^; HRESIMS calcd. For C_45_H_67_N_7_O_8_Na [M + Na]^+^ 856.4949, found 856.4991.

HO-Hmp-Leu-*N*-Me-d-Gln-Ile-Gly-*N*-Me-d-Phe-NH-TIQ (**C8**): Yield 59%; *R*_f_ (CHCl_3_/MeOH 20:1) 0.22; 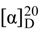 = −15.1 (*c* 0.04 MeOH); ^1^H-NMR (CDCl_3_, 600 MHz) δ 7.24–7.04 (m, 11H), 5.95 (brs, 1H), 5.76 (dd, *J* = 15.4, 7.7 Hz, 1H), 5.70 (brs, 1H), 5.07 (t, *J* = 7.5 Hz, 1H), 4.95 (dd, *J* = 14.5, 7.1 Hz, 1H), 4.71 (d, *J* = 17.2 Hz, 1H), 4.62 (d, *J* = 16.8 Hz, 1H), 4.58 (s, 1H), 4.30 (t, *J* = 7.0 Hz, 1H), 4.04 (dd, *J* = 17.4, 4.9 Hz, 1H), 4.01–3.96 (m, 1H), 3.92 (dd, *J* = 4.0 Hz, 1H), 3.80 (dd, *J* = 17.6, 3.7 Hz, 1H), 3.59–3.54 (m, 1H), 3.29 (dd, *J* = 13.7, 8.6 Hz, 1H), 3.03 (s, 3H), 3.00 (s, 3H), 2.97–2.89 (m, 1H), 2.87–2.81 (m, 1H), 2.79–2.74 (m, 1H), 2.32 (td, *J* = 13.9, 7.0 Hz, 1H), 2.26-2.20 (m, 1H), 2.19–2.14 (m, 1H), 1.99 (td, *J* = 14.3, 7.7 Hz, 1H), 1.84–1.80 (m, 2H), 1.63–1.57 (m, 3H), 1.48–1.41 (m, 2H), 1.26–1.19 (m, 1H), 1.17–1.09 (m, 1H), 0.98–0.94 (m, 9H), 0.91–0.85 (m, 9H); ^13^C-NMR (CDCl_3_, 150 MHz) δ 174.5, 174.3, 174.1, 171.5, 171.3, 169.7, 168.7, 168.3, 167.7, 167.4, 136.6, 134.3, 134.0, 132.8, 132.7, 129.1, 128.9, 128.6, 128.5, 128.4, 127.1, 126.9, 126.6, 126.5, 126.3, 125.6, 76.5, 57.8, 56.3, 54.6, 53.9, 47.9, 44.7, 43.1, 41.3, 41.2, 41.1, 41.0, 38.3, 37.1, 35.8, 35.5, 32.2, 31.0, 29.9, 29.7, 29.5, 29.3, 28.1, 24.9, 24.8, 23.8, 23.1, 23.0, 22.1, 15.6, 11.8, 11.3; ESI-MS *m/z*: 834.3 [M + H]^+^, 856.3 [M + Na]^+^; HRESIMS calcd. for C_45_H_67_N_7_O_8_Na [M + Na]^+^ 856.4949, found 856.4977.

HO-Hmp-Leu-*N*-Me-Ala-Ile-Gly-*N*-Me-d-Phe-NH-Bn (**C9**): Yield 63%; *R*_f_ (CHCl_3_/MeOH 20:1) 0.42; 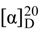 = −20.2 (*c* 0.1 MeOH); ^1^H-NMR (dimethyl sulfoxide-*d*_6_ (DMSO-*d*_6_), 600 MHz) δ 8.69 (t, *J* = 5.9 Hz, 1H), 7.91 (br, 1H), 7.67 (br, 1H), 7.50 (br, 1H), 7.30–7.10 (m, 10H), 5.20–5.18 (m, 1H), 4.98–4.96 (m, 1H), 4.80–4.77 (m, 1H), 4.30–4.27 (m, 2H), 4.20–4.16 (m, 1H), 4.00–3.96 (m, 1H), 3.70–3.67 (m, 2H), 3.25–3.18 (m, 1H), 2.92–2.88 (m, 1H), 2.90 (s, 3H), 2.87 (s, 3H), 1.70–1.66 (m, 2H), 1.57–1.55 (m, 1H), 1.45–1.42 (m, 1H), 1.36–1.31 (m, 2H), 1.27–1.23 (m, 1H), 1.20–1.18 (m, 3H), 1.14–0.97 (m, 2H), 0.90–0.74 (m, 18H); ^13^C-NMR (DMSO-*d*_6_, 150 MHz) δ 173.7, 173.6, 171.9, 171.1, 170.8, 169.3, 138.1, 136.8, 129.0, 128.7, 128.6, 127.6, 127.4, 126.9, 76.4, 58.6, 58.1, 57.6, 47.5, 43.4, 41.6, 41.5, 38.9, 37.2, 37.0, 34.8, 29.8, 24.9, 24.7, 23.6, 23.5, 22.0, 21.5, 15.7, 15.6, 11.9, 11.5; HRESIMS calcd. for C_41_H_62_N_6_O_7_Na [M + Na]^+^ 773.4578, found 773.4577.

#### 3.1.10. General Procedure for the Preparation of Compounds **N1**–**N2**

Prepared by following similar method used for **C1**–**C9**, but using *n*-pentanoic acid or 2-hydroxyacetic acid to block the *N*-terminus.

*n*-Pentanoyl-Leu-*N*-Me-d-Gln-Ile-Gly-*N*-Me-d-Phe-NH-Bn (**N1**): Yield 17%; *R*_f_ (CHCl_3_/MeOH 10:1) 0.61; 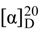 = −21.8 (*c* 0.10, CHCl_3_); ^1^H-NMR (DMSO-*d*_6_, 600 MHz) δ 8.63 (br, 1H), 8.10 (br, 1H), 7.95 (br, 1H), 7.80 (br, 1H), 7.28–7.17 (m, 11H), 6.75–6.72 (m, 1H), 5.22–5.18 (m, 1H), 4.90–4.85 (m, 1H), 4.29–4.17 (m, 3H), 4.04–4.00 (m, 1H), 3.70–3.66 (m, 1H), 3.50–3.47 (m, 1H), 3.20–3.17 (m, 1H), 2.93–2.71 (m, 6H), 2.58–2.55 (m, 1H), 2.31–1.88 (m, 6H), 1.77–1.73 (m, 2H), 1.50–1.23 (m, 8H), 0.93–0.76 (m, 15H); ^13^C-NMR (DMSO-*d*_6_, 150 MHz) δ 174.4, 173.1, 172.9, 170.1, 169.4, 169.2, 169.0, 139.7, 138.4, 129.3, 128.7, 128.6, 127.7, 127.5, 127.1, 58.7, 47.9, 42.9, 42.8, 41.4, 41.0, 35.4, 34.6, 31.9, 31.4, 29.6, 29.3, 28.0, 27.9, 24.8, 23.5, 22.3, 22.1, 21.9, 15.8, 14.5, 14.2, 11.5; HRESIMS calcd. for C_42_H_64_N_7_O_7_ [M + H]^+^ 778.4867, found 778.4860.

2-Hydroxyacetyl-Leu-*N*-Me-d-Gln-Ile-Gly-*N*-Me-d-Phe-NH-Bn (**N2**): Yield 26%; *R*_f_ (CHCl_3_/MeOH 10:1) 0.62; 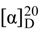 = −9.3 (*c* 0.11, CHCl_3_); ^1^H-NMR (CDCl_3_, 600 MHz) δ 7.64 (br, 1H), 7.45 (br, 2H), 7.36 (br, 1H), 7.16–7.03 (m, 10H), 6.92–6.88 (m, 2H), 5.16–5.12 (m, 1H), 4.80–4.76 (m, 2H), 4.49–4.37 (m, 2H), 4.21–4.17 (m, 1H), 4.08–3.90 (m, 2H), 3.76–3.58 (m, 2H), 3.30–3.26 (m, 1H), 2.97–2.95 (m, 1H), 2.88 (s, 3H), 2.81 (s, 3H), 2.25–2.10 (m, 3H), 1.66–1.34 (m, 3H), 1.29–1.03 (m, 4H), 0.90–0.63 (m, 12H); HRESIMS calcd. for C_39_H_58_N_7_O_8_ [M + H]^+^ 752.4347, found 752.4344.

#### 3.1.11. General Procedure for the Preparation of Compounds **13a**–**c**

Compound **11** or **12** (0.2 mmol) was treated with HCl/EtOAc (4 mol/L, 2 mL) for 1 h then concentrated *in vacuo*. The residue was redissolved in EtOAc (10 mL) and concentrated *in vacuo* again. The resulting solid was dried under vacuum for 2 h and then dissolved in 8 mL of dry DCM-DMF (3:1). After being cooled with an ice-water bath for 10 min, Fmoc-*N*-Me-d-Gln-OH (0.2 mmol) or Boc-d-Gln-OH (0.2 mmol), EDC (0.24 mmol), HOAt (0.24 mmol) and NaHCO_3_ (0.3 mmol) were added consecutively. The mixture was stirred at this temperature for 2 h and then at rt for another 12 h. The mixture was diluted with 80 mL of EtOAc and washed with 10% citric acid, 5% NaHCO_3_, water and brine. The organic layer was dried over Na_2_SO_4_, then concentrated *in vacuo*. The residue was purified by flash column chromatography to give the desired compounds **13a**–**c**. These three compounds were used directly without further structural characterizations.

#### 3.1.12. General Procedure for the Preparation of Compounds **M1**–**M3**

Compounds **13a**~**c** (0.1 mmol) were treated with HCl/EtOAc (4 mol/L, 2 mL) (**13a**, **13c**) or DEA/DCM (**13b**) for 1 h, then concentrated *in vacuo*. The residue was redissolved in EtOAc (10 mL) and concentrated again. The resulting solid was dried under vacuum for 2 h and then dissolved in 8 mL of dry DCM-DMF (3:1). After being cooled with an ice-water bath for 10 min, Hmp-Leu-OH (0.1 mmol), EDC (0.12 mmol), HOAt (0.12 mmol) and NaHCO_3_ (0.2 mmol) were added consecutively. The mixture was stirred at this temperature for 2 h and then at rt for another 12 h. The mixture was diluted with 80 mL of EtOAc and washed with 10% citric acid, 5% NaHCO_3_, water and brine. The organic layer was dried over Na_2_SO_4_, then concentrated *in vacuo*. The residue was purified by flash column chromatography to give the desired compounds **M1**–**M3**.

HO-Hmp-Leu-d-Gln-Ile-Gly-*N*-Me-d-Phe-NH-Bn (**M1**): Yield 75%; *R*_f_ (CHCl_3_/MeOH 10:1) 0.53; 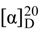 = −3.7 (*c* 0.11, MeOH); ^1^H-NMR (DMSO-*d*_6_, 600 MHz) δ 8.70 (t, *J* = 5.9 Hz, 1H), 8.22 (br, 1H), 7.93 (t, *J* = 5.0 Hz, 1H), 7.71 (d, *J* = 9.4 Hz, 1H), 7.60 (d, *J* = 8.7 Hz, 1H), 7.30–7.14 (m, 11H), 6.75–6.73 (m, 1H), 5.25–5.21 (m, 1H), 4.45–4.43 (m, 1H), 4.40–4.17 (m, 4H), 4.01–3.97 (m, 1H), 3.77–3.72 (m, 1H), 3.46–3.40 (m, 1H), 3.24 (dd, *J* = 14.6, 5.9 Hz, 1H), 2.95–2.90 (m, 1H), 2.87 (s, 3H), 2.12–2.09 (m, 2H), 1.84–1.80 (m, 1H), 1.70–1.64 (m, 3H), 1.55–1.51 (m, 1H), 1.49–1.33 (m, 3H), 1.25–1.21 (m, 1H), 1.16–1.01 (m, 3H), 0.88–0.76 (m, 18H); ^13^C-NMR (DMSO-*d*_6_, 150 MHz) δ 174.4, 173.4, 172.5, 171.4, 170.2, 169.5, 169.0, 139.9, 138.5, 129.7, 128.9, 128.8, 128.7, 127.8, 127.5, 127.2, 126.8, 75.6, 58.7, 57.2, 52.6, 50.8, 42.7, 41.4, 38.7, 37.5, 34.7, 32.0, 31.5, 28.1, 24.7, 24.6, 23.8, 23.6, 22.2, 16.1, 15.8, 12.3, 11.7; HRESIMS calcd. For C_42_H_64_N_7_O_8_ [M + H]^+^ 794.4816, found 794.4801.

HO-Hmp-Leu-*N*-Me-d-Gln-Ile-Gly-d-Phe-NH-Bn (**M2**): Yield 35%; *R*_f_ (CHCl_3_/MeOH 10:1) 0.50; 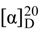 = −34.7 (*c* 0.11, MeOH); ^1^H-NMR (DMSO-*d*_6_, 600 MHz) δ 8.43 (t, *J* = 5.8 Hz, 1H), 8.27 (t, *J* = 6.1 Hz, 1H), 8.13 (br, 1H), 7.68 (d, *J* = 8.2 Hz, 1H), 7.62 (d, *J* = 8.3 Hz, 1H), 7.28–7.18 (m, 10H), 7.13–7.09 (m, 2H), 4.94–4.90 (m, 1H), 4.77–4.72 (m, 1H), 4.51–4.44 (m, 1H), 4.31–4.27 (m, 1H), 4.21–4.18 (m, 1H), 4.11–4.07 (m, 1H), 3.77–3.71 (m, 2H), 3.62–3.59 (m, 1H), 3.05–3.01 (m, 1H), 2.94 (s, 3H), 2.83–2.79 (m, 1H), 1.82–1.77 (m, 2H), 1.71–1.62 (m, 5H), 1.38–1.23 (m, 3H), 1.18–1.08 (m, 2H), 1.05–0.98 (m, 1H), 0.93–0.76 (m, 18H); ^13^C-NMR (DMSO-*d*_6_, 150 MHz) δ 175.1, 174.1, 173.4, 171.9, 171.4, 170.6, 169.1, 139.6, 138.3, 129.7, 128.8, 128.7, 127.6, 127.2, 126.9, 73.0, 57.7, 56.0, 54.9, 47.3, 43.2, 42.6, 42.5, 41.7, 38.7, 38.2, 37.0, 32.1, 29.6, 24.8, 24.4, 23.8, 23.7, 21.9, 16.0, 15.8, 12.2, 11.5; HRESIMS calcd. for C_42_H_64_N_7_O_8_ [M + H]^+^ 794.4816, found 794.4782.

HO-Hmp-Leu-d-Gln-Ile-Gly-d-Phe-NH-Bn (**M3**): Yield 32%; *R*_f_ (CHCl_3_/MeOH 10:1) 0.51; 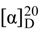 = 46.0 (*c* 0.1, MeOH); ^1^H-NMR (DMSO-*d*_6_, 600 MHz) δ 8.4 (t, *J* = 5.9 Hz, 1H), 8.28 (t, *J* = 5.5 Hz, 1H), 8.22 (d, *J* = 7.8 Hz, 1H), 8.15 (br, 1H), 7.85 (d, *J* = 6.6 Hz, 1H), 7.58 (d, *J* = 8.7 Hz, 1H), 7.28–7.17 (m, 10H), 7.13 (d, *J* = 7.3 Hz, 2H), 4.55–4.50 (m, 1H), 4.48–4.42 (m, 1H), 4.34–4.18 (m, 3H), 4.11–4.02 (m, 1H), 3.79–3.72 (m, 2H), 3.60–3.55 (m, 1H), 3.03–2.99 (m, 1H), 2.85–2.79 (m, 1H), 2.15–2.04 (m, 2H), 1.86–1.83 (m, 1H), 1.73–1.66 (m, 3H), 1.53–1.49 (m, 1H), 1.48–1.41 (m, 3H), 1.34–1.32 (m, 1H), 1.14–1.02 (m, 2H), 0.86–0.75 (m, 18H); ^13^C-NMR (DMSO-*d*_6_, 150 MHz) δ 174.5, 173.4, 172.6, 172.2, 171.9, 171.4, 169.2, 139.6, 138.4, 129.7, 128.8, 127.5, 127.2, 126.9, 75.5, 57.7, 55.4, 55.1, 52.7, 50.7, 42.6, 42.2, 38.7, 38.1, 37.0, 32.1, 27.9, 24.8, 24.7, 23.8, 23.6, 22.1, 16.0, 15.7, 12.3, 11.6; HRESIMS calcd. for C_41_H_62_N_7_O_8_ [M + H]^+^ 780.4660, found 780.4648.

### 3.2. Cytotoxicity Assays

All of the prepared analogues (**S1**–**S4**, **C1**–**C9**, **N1**–**N2**, **M1**–**M3**) were tested for their *in vitro* anticancer activities against KB and A549 cell lines. Briefly, cells were seeded into 96-well plates and cultured overnight, treated with tested samples for 72 h. Then, the cells were fixed with 10% trichloroacetic acid (100 µL) and stained with sulforhodamine B (Sigma, St. Louis, MO, USA). The sulforhodamine B (100 µL) in the cells was dissolved in 10 mmol/L Tris-HCl (150 µL) and measured at 515 nm using a multiwell spectrophotometer (VERSAmax, Molecular Devices, Sunnyvale, CA, USA). The inhibition rate on cell proliferation was calculated for each well as (A515_control cells_ − A515_treated cells_)/A515_control cells_ × 100% (A515: optical density value at 515 nm). The average IC_50_ values were determined by the Logit method from at least three independent tests.

## 4. Conclusions

In conclusion, eighteen analogues of tasiamide were designed and synthesized to investigate the structure-activity relationship (SAR) of this marine linear peptide. The result indicated that a certain length of the peptide chain might be essential for cytotoxicity. Modifications on the *C*-terminus with aromatic groups were tolerated. On the other hand, simplifications on the Hmp residue or modifications on the *N*-methylated amino acids led to inactive analogues. Further structural optimization and SAR research are ongoing in our laboratory and will be reported in the future.
